# Time-dependent diffusion MRI in determining mismatch repair status of endometrial cancer

**DOI:** 10.1186/s41747-026-00830-y

**Published:** 2026-11-03

**Authors:** Jiarui Zhang, Diwei Shi, Jing Xi Joshua Li, Cheuk Nam Hwang, Fan Liu, Zongshu Wang, Grace Ho, Thorsten Feiweier, Juan Wei, Hua Guo, Elaine YP Lee

**Affiliations:** 1 Department of Diagnostic Radiology, School of Clinical Medicine, Li Ka Shing Faculty of Medicine, University of Hong Kong, Hong Kong, China; 2 Tsinghua Shenzhen International Graduate School, Tsinghua University, Shenzhen, China; 3 Department of Pathology, School of Clinical Medicine, Li Ka Shing Faculty of Medicine, University of Hong Kong, Hong Kong, China; 4 Center for Biomedical Imaging Research, School of Biomedical Engineering, Tsinghua University, Beijing, China; 5 Department of Radiology, Queen Mary Hospital, Hong Kong, China; 6 MR Research and Clinical Translation, Siemens Healthineers AG, Erlangen, Germany; 7 MR Research Collaboration, Siemens Healthineers, Hong Kong, China

**Keywords:** Biomarkers, Diffusion magnetic resonance imaging, DNA mismatch repair, Endometrial neoplasms, Preoperative care

## Abstract

**Objectives:**

Mismatch repair (MMR) proteins are important in determining therapeutic strategy in endometrial cancer (EC). We investigated the role of microstructural parameters derived from time-dependent diffusion magnetic resonance imaging (td-dMRI) in differentiating MMR status of EC.

**Materials and methods:**

This prospective single-center study enrolled patients with suspected EC who underwent preoperative td-dMRI. Tumors were classified as MMR-deficient (dMMR) and MMR-proficient (pMMR) by immunohistochemistry. The microstructural parameters, including intracellular volume fraction (${v}_{{in}}$), cell diameter (d), cellularity (${{\rm{\rho }}}$), extracellular diffusivity (${D}_{{ex}}$), and water exchange rate constant (${k}_{{in}}$), were derived by fitting td-dMRI data with three methods (IMPULSED, JOINT, and EXCHANGE), together with the apparent diffusion coefficient (ADC). Differences in these microstructural parameters between dMMR and pMMR EC were compared. The diagnostic performances were evaluated by the areas under the receiver operating characteristic curve (AUC).

**Results:**

Eighty-two patients (17 dMMR, 61.8 ± 10.1 years and 65 pMMR, 58.6 ± 11.8 years) were recruited. dMMR EC exhibited higher ${v}_{{in}}$, ${{\rm{\rho }}}$ and lower ${D}_{{ex}}$, ADC values than pMMR EC (*p* ≤ 0.003). IMPULSED and JOINT demonstrated higher AUCs than conventional ADC (*p* = 0.025 and 0.046, respectively). The combined selected microstructural parameters (${v}_{{\mathrm{in}}}$ and ${D}_{{ex}}$) derived from JOINT achieved an AUC of 0.903; JOINT demonstrated superior parametric stability and marginally better calibration than IMPULSED.

**Conclusion:**

In this exploratory study, td-dMRI discriminated between dMMR and pMMR ECs, providing information on the intricate microscopic organizational structures in tumors. The microstructural parameters may serve as a promising method to improve the diagnostic efficacy of diffusion-weighted MRI in differentiating MMR status of EC.

**Key Points:**

***Question*** There remains an unmet need for noninvasive, preoperative tools to assess DNA MMR status in EC.

***Findings*** td-dMRI showed significant differences in microstructural parameters between dMMR and pMMR tumors.

***Relevance statement*** td-dMRI shows potential as a noninvasive imaging approach for preoperative assessment of MMR status in EC and may support individualized treatment planning.

**Graphical Abstract:**

**Figure:**
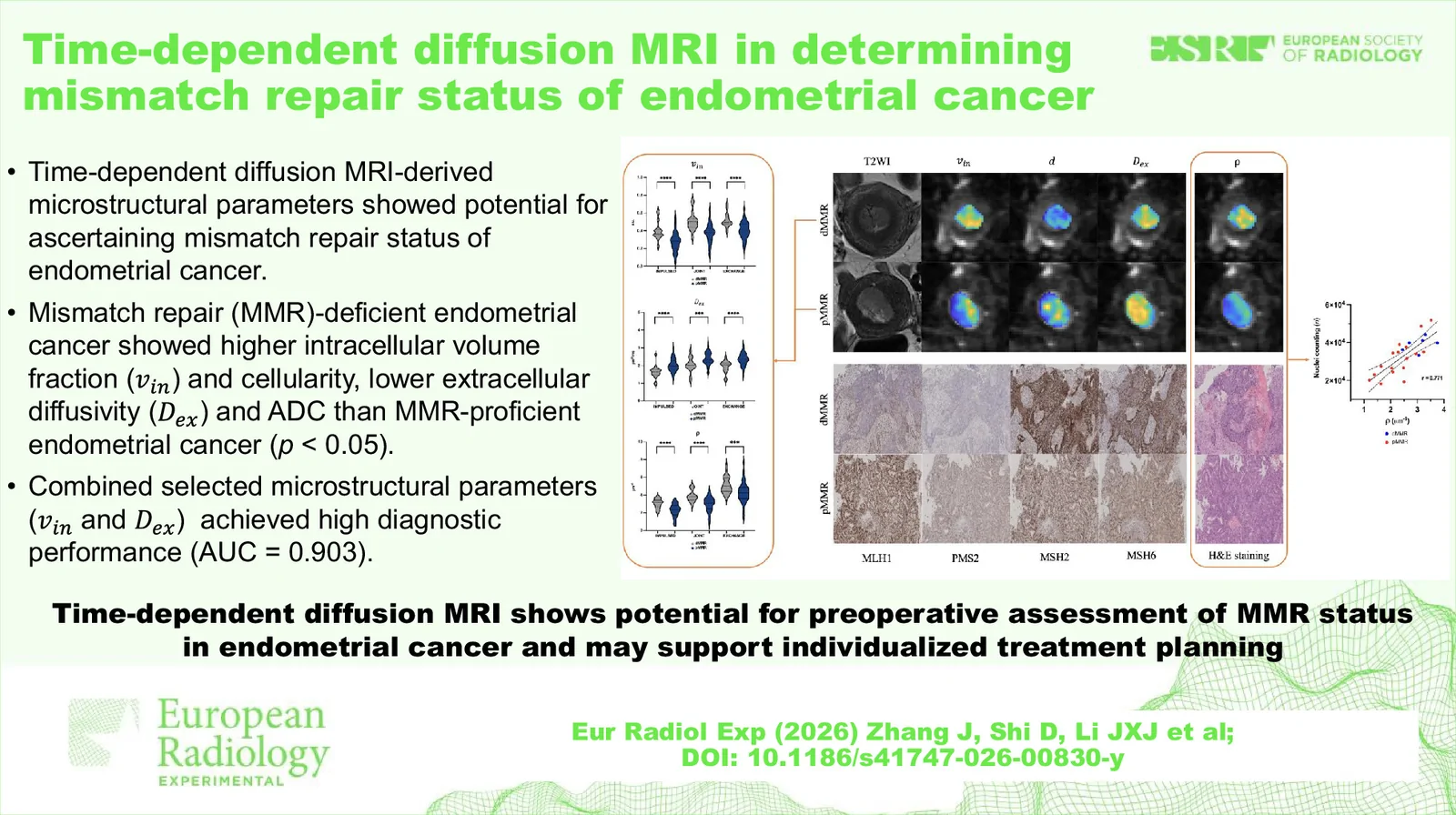

## Introduction

Endometrial cancer (EC) is the most common gynecological cancer in developed countries. The global incidence of EC is expected to rise, which is associated with the increased prevalence of obesity, metabolic syndrome, and an ageing population [1]. The Cancer Genome Atlas provides a comprehensive molecular classification that has demonstrated superior performance over conventional histological classification in predicting outcomes and guiding treatment in EC, particularly in the application of targeted therapies and immunotherapy [2–4]. Mismatch repair (MMR) proteins, including MLH1, PMS2, MSH2, and MSH6, play crucial roles in the ability of cells to repair errors during DNA replication, categorizing tumors as MMR-deficient (dMMR) or MMR-proficient (pMMR) [5]. Representing approximately 20–30% of all ECs [6], patients with dMMR exhibit an intermediate prognostic risk, reflecting their unique clinical characteristics with special therapeutic implications [7–9]. Additionally, dMMR is present in over 90% of patients with EC in Lynch syndrome [10]. Of note, the expression of programmed death-ligand 1 (PD-L1) is increased in dMMR tumors [11]. Previous studies have investigated the potential predictive value of dMMR testing in response to immunotherapy [12, 13]. A phase II trial demonstrated durable anti-tumor activity and a favorable safety profile in advanced dMMR EC, supporting durvalumab as a standalone immunotherapy for dMMR EC [12].

Beyond their clinical and molecular distinctions, dMMR ECs exhibit characteristic microstructural alterations of the tumor microenvironment. They are characterized by dense tumor-infiltrating lymphocyte infiltration and stromal remodeling, resulting in a more heterogeneous and denser cellular microenvironment compared with pMMR EC [14]. These immune-driven microstructural differences, including increased intracellular volume fraction and altered extracellular diffusivity, provide a biological rationale for the hypothesis that imaging techniques sensitive to tissue microstructure at the cellular scale may noninvasively reflect MMR status in EC.

Currently, the MMR status of EC is commonly determined by immunohistochemistry (IHC) on the available tissue samples after surgery and lacks a noninvasive preoperative prediction method. Liquid biopsy has been investigated but remains at an exploratory stage and has not yet been widely adopted in clinical practice [15]. Radiomics from routinely acquired computed tomography [16, 17] and magnetic resonance imaging (MRI) [18, 19] have shown encouraging performance for predicting MMR status and offer the practical advantage of using readily available standard clinical sequences. However, radiomic features, including those derived from standard diffusion-weighted imaging (DWI), are largely data-driven and lack a direct biological correlate, which may limit their interpretability. Therefore, there remains a need for rigorous and clinically validated noninvasive methods to predict MMR status preoperatively while reflecting the underlying tumor microstructure.

DWI is valuable in evaluating EC by measuring water molecular mobility within tissue [20]. Conventional DWI uses a pulsed gradient spin-echo (PGSE) sequence with relatively long diffusion time, typically more than 20 ms. The recently developed time-dependent diffusion MRI (td-dMRI), including oscillating gradient spin-echo diffusion (OGSE) sequence, is capable of reducing diffusion time to less than 10 ms by applying oscillating waveforms. It holds potential for noninvasively detecting more intricate microscopic organizational structures with the ultra-short diffusion time [21]. This technology incorporates signal acquisitions under different diffusion times, decouples DWI signals, and quantifies microstructural features based on multi-compartmental biophysical models. This approach allows the mapping of tumor microenvironment by calculating several microstructural parameters, including intracellular volume fraction (${v}_{{in}}$), cell diameter (d), cellularity or cell density $({{\rm{\rho }}})$, extracellular diffusivity (${D}_{{ex}}$), and water exchange rate constant $({k}_{{in}})$ that highlight time-dependent diffusion properties [22]. Several studies have demonstrated the potential of td-dMRI in prostate cancer [22] and breast cancer [23]. At present, only a few researchers have explored the utilization of apparent diffusion coefficient (ADC) values obtained from OGSE sequences and td-dMRI in EC for risk stratification based on the traditional histological classification [24, 25]. Given the microstructural differences between dMMR and pMMR EC outlined above, td-dMRI is therefore well positioned to bridge this gap by providing biophysically interpretable, microstructure-level parameters directly relevant to the underlying tumor biology.

We hypothesized that td-dMRI would discriminate dMMR and pMMR ECs and enhance the diagnostic performance in determining MMR status of EC noninvasively.

## Materials and methods

This prospective study was approved by the local Institutional Review Board (UW24-561, UW25-244) and adhered to the Declaration of Helsinki. Informed written consent was obtained for all patients.

### Study population

Patients were consecutively recruited between September 2024 and June 2025. All patients fulfilled the following inclusion and exclusion criteria. Inclusion criteria: (1) histologically confirmed EC; (2) availability of pretreatment pelvic MRI with DWI; and (3) no other malignancies in the pelvis, except EC. Exclusion criteria: (1) poor image quality; (2) tumor lesion with less than 20 mm^2^ per slice (it corresponded to fewer than five voxels given the in-plane resolution of 2.0 × 2.0 mm², which was considered insufficient for reliable multiparameter model fitting).

### MMR IHC and histopathologic analysis

All MMR IHC staining and surgically resected specimens were obtained within 1 month after MRI and examined by a board-certified pathologist with over 10 years of experience and discussed at the weekly multidisciplinary meetings. The expression of four MMR proteins (MLH1, PMS2, MSH2, and MSH6) was detected by IHC using the BenchMark ULTRA IHC/ISH system (Roche Diagnostics, Basel, Switzerland) on 4-micron thick FFPE sections, with the antibodies MLH1 (G168-15), MSH2 (FE11), MSH6 (44), and PMS2 (A16-4) according to manufacturer’s protocols. The intact expression of all four MMR proteins was classified as pMMR, while the loss of at least one MMR protein was classified as dMMR [6]. All slides were digitized using NanoZoomer S210 (Hamamatsu Photonics, Hamamatsu, Japan). The cell nuclei were calculated using QuPath software (Version 0.5.1) on hematoxylin and eosin-stained images (magnification: 40×; area: 4 × 10^6^ µm^2^). Histopathological correlation was performed in a subset of 23 patients for whom digitized whole-slide images of sufficient quality were available. As whole-slide digitization of hematoxylin and eosin slides was not part of the routine pathology workflow at our institution, slides were retrospectively digitized only for cases in which the original sections met the clarity and quality requirements for reliable digital nuclear segmentation.

### Image acquisition

Preoperative MRI examinations were performed on two 3.0-T MRI scanners (MAGNETOM Skyra and Vida, Siemens Healthineers AG, Erlangen, Germany), with maximum gradients of 45/60 mT/m and a maximum slew rate of 200 mT/m/ms. MRI was acquired with the patient in the supine position using an external pelvic phased-array coil. An advanced diffusion-weighted MRI work-in-progress sequence provided by Siemens was employed to acquire OGSE with the frequencies of 20/40 Hz and PGSE data. The following parameters were used for both sequences: repetition time/echo time = 5,100/130 ms; field of view = 300 × 224 mm^2^; in-plane resolution = 2 × 2 mm^2^; number of slices = 20; and slice thickness = 4 mm. The acquisition time of the diffusion-weighted MRI protocol was approximately 8 min. The detailed parameters are listed in Table 1. The other routine sequences included axial T1-weighted imaging, axial T2-weighted imaging, and three-dimensional contrast-enhanced T1-weighted imaging (Supplementary Table S1).

**Table 1:** Parameters of diffusion-weighted MRI

Sequence	δ (ms)	Δ (ms)	Cycles	*f* (Hz)	*t*_*diff*_ (ms)	b (s/mm^2^)	Scan time
PGSE	12	74	-	-	70	0, 250, 500, 750, 1,000, 1,200, 1,400	3 min 51 s
OGSE	50.9	62.5	1	20	12.73	0, 250, 500, 750, 1,000	1 min 51 s
	50.9	62.5	2	40	6.36	0, 250	2 min 2 s

*δ* Duration of each diffusion gradient, *Δ* Separation between two gradients, *f* Frequency, *MRI* Magnetic resonance imaging, *OGSE* Oscillating gradient spin-echo, *PGSE* Pulsed gradient spin-echo, ${t}_{{diff}}$ Diffusion time

### Image analysis

#### Preprocessing

Alignment between the three diffusion-weighted sequences (PGSE, OGSE at 20 and 40 Hz) was performed using the built-in “imregtform” and “imwarp” functions of MATLAB R2022b (MathWorks). Four-dimensional MRI denoising (length, width, height, and *b*-value) was performed using Marchenko-Pastur principal component analysis [26] by exploiting data redundancy in the principal component analysis domain using the universal properties of the eigenspectrum of random covariance matrices.

#### Data fitting

Three methods, IMPULSED [27], JOINT [28] and EXCHANGE [29], were used to analyze the td-dMRI signals acquired at different diffusion time (PGSE: the diffusion time is 70 ms and the range of *b*-value is 0–1,400 s/mm^2^; OGSE-20Hz: 12.73 ms and 0–1,000 s/mm^2^; OGSE-40Hz: 6.36 ms and 0–250 s/mm^2^). IMPULSED models the tumor cell as an impermeable sphere and ignores water exchange between the intra- and extracellular compartments. Three free microstructural parameters are measured: ${v}_{{in}}$, d, ${D}_{{ex}}$. In contrast, JOINT and EXCHANGE incorporate transcytolemmal water exchange into the biophysical modeling and provide an additional free parameter, ${k}_{{in}}$, which describes the water exchange rate between intra- and extracellular compartments. Compared to JOINT, EXCHANGE has made several refinements in the biophysical modeling, providing more accurate estimations of microstructural features. More information on the three quantitative methods is detailed in Supplementary Appendix S1.

The microstructural parameters derived from IMPULSED, JOINT, and EXCHANGE were all calculated using the data-fitting platform MATI [30]. Note that the intracellular intrinsic diffusivity was fixed at 1.58 μm^2^/ms to stabilize the fitting results as reported previously [31]. The image-derived cellularity index ρ was calculated by ${v}_{{in}}/d\times 100\,$[22]. Three time-dependent ADC metrics, ADC_PGSE_, ADC_20Hz_, and ADC_40Hz_, corresponding to PGSE, OGSE-20Hz, and OGSE-40Hz, respectively, were calculated by fitting the mono-exponential decay model: $S={S}_{0}\exp (-b\cdot {\mathrm{ADC}})$.

#### Volume of interest (VOI)

A postgraduate student with medical training was guided in tumor segmentation and manually segmented all the EC tumors on diffusion-weighted MRI. The VOI was placed to encompass the entire tumor on the PGSE images (*b*  =  1,400 s/mm^2^), with reference to T2-weighted images, using ITK-SNAP software (Version 4.2.2, www.itksnap.org). Surrounding muscle and necrotic tissue were excluded from the VOIs. These VOIs were subsequently verified by a board-certified radiologist with more than 15 years of experience in pelvic imaging and a special interest in gynecological imaging. Both were blinded to the clinicopathological information, except for the diagnosis of EC. The time-dependent ADC values and model-fitted microstructural parameters were calculated within each voxel and then averaged over the whole VOI. Inter- and intra-reader agreement assessments were conducted (Supplementary Appendix S2).

### Statistical analysis

Inter-and intra-reader agreements of parameter measurements were assessed using the intraclass correlation coefficient (ICC). ICC values were interpreted as poor (< 0.50), moderate (0.50 ≤ ICC < 0.75), good (0.75 ≤ ICC ≤ 0.90), or excellent (> 0.90). The Pearson correlation coefficient was used to assess the correlation between cellularity ${{\rm{\rho }}}$ derived from td-dMRI and histopathological results. The Mann–Whitney *U*-test was performed to assess the differences in the imaging parameters of td-dMRI between dMMR and pMMR EC, including microstructural parameters and time-dependent ADC values. Logistic regression evaluated the diagnostic performance of the different diffusion-weighted MRI imaging metrics in determining the MMR status. The area under the receiver-operating characteristic curve (AUC) was calculated to assess the discriminative ability of single or combined parameters. The indicators of accuracy, sensitivity, specificity, positive predictive value, and negative predictive value were also presented. Multivariable regression was performed with variables chosen with a stepwise procedure based on likelihood-ratio tests, adding predictors one at a time in the order of statistical significance. Subsequently, the DeLong test was employed to compare the AUCs of the combination models with those of single-parameter models. Internal validation was performed using bootstrap resampling (1,000 iterations) with Harrell’s optimism correction and calibration assessment to evaluate potential overfitting given the limited events per variable. To assess potential influence of inter-scanner variability, a sensitivity analysis was performed by including scanner type as an additional covariate in each combination model. The statistical significance level was set at two-tailed *p*  <  0.05 for all analyses. Statistical analyses were conducted using commercial statistical software (Prism 10, GraphPad Software, Boston, MA, USA; SPSS 28.0, IBM, Armonk, NY, USA).

## Results

### Study population

**Fig. 1:**
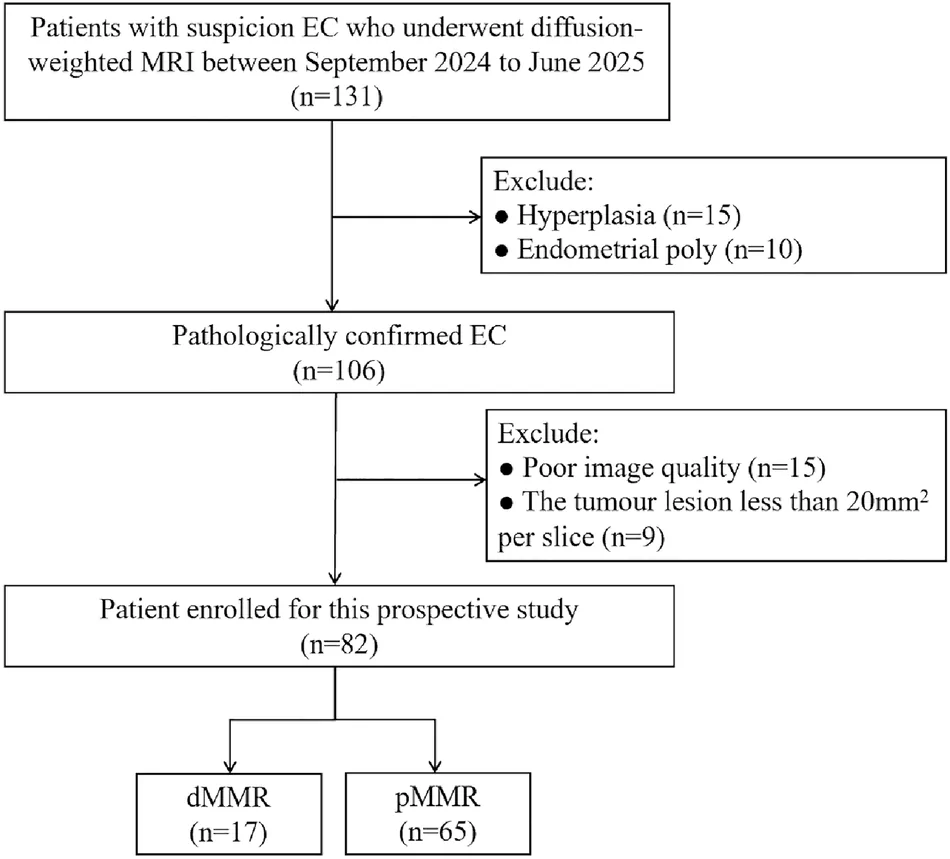
Patient enrollment workflow chart. dMMR, Mismatch repair deficient; EC, Endometrial cancer; MRI, Magnetic resonance imaging; pMMR, Mismatch repair proficient

**Fig. 2:**
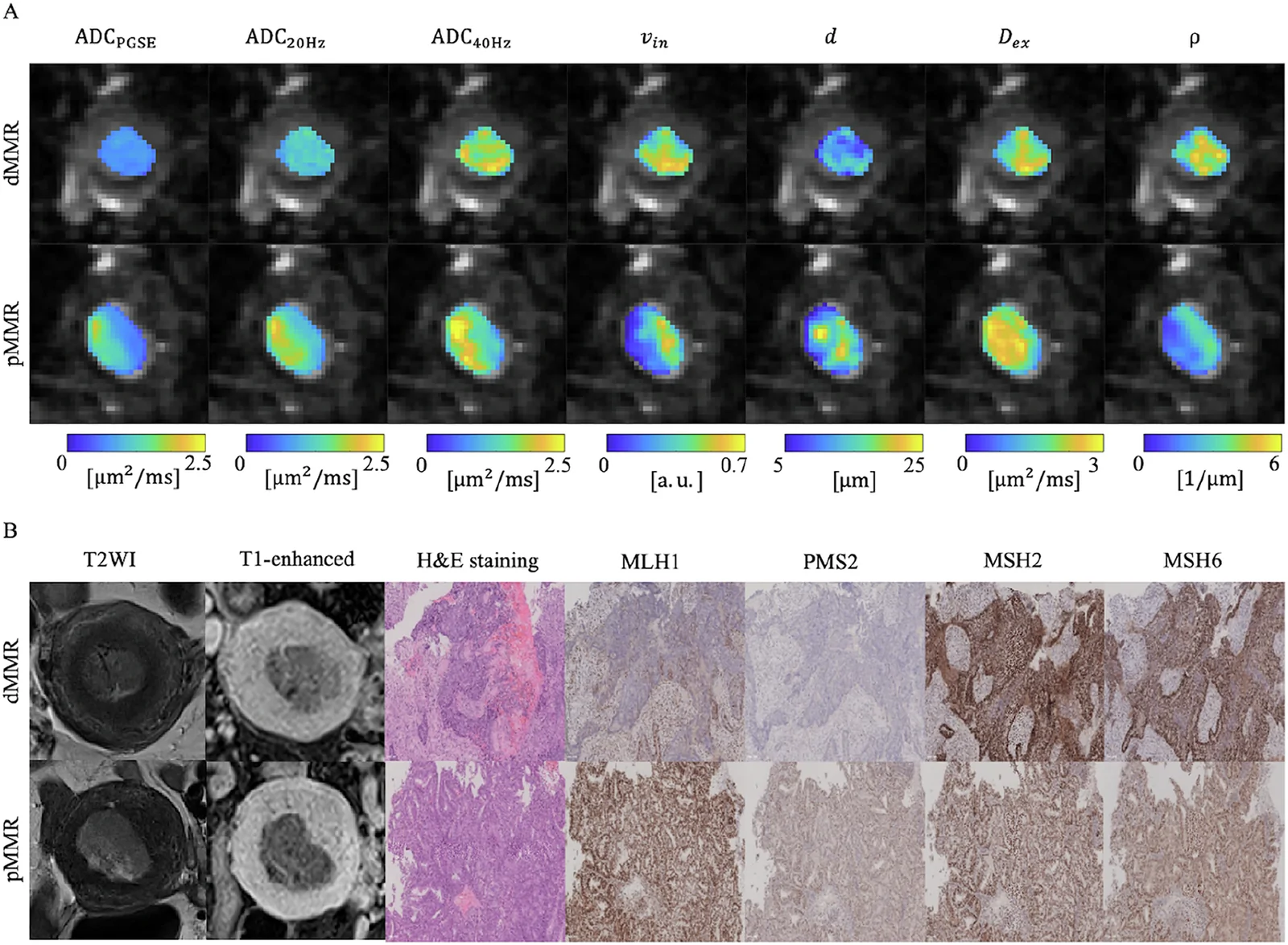
Two exemplars of dMMR EC (a 59-year-old woman) and pMMR EC (a 73-year-old woman) with microstructural feature maps, corresponding Hematoxylin and eosin staining and IHC analyses. **A** IMPULSED-derived microstructural feature maps, including ADC maps, ${v}_{{in}}$, d, ${D}_{{ex}}$ and ${{\rm{\rho }}}$ for dMMR EC (upper panel) and pMMR EC (lower panel). **B** T2WI, T1-enhanced, and histopathological staining images. Brown stain is intact nuclear staining, while blue counterstains indicate absent or negative staining. IHC analyses of dMMR EC indicated the loss of MLH1 and PMS2 with intact expression of MSH2 and MSH6 (upper panel), whereas intact nuclear staining of MLH1, PMS2, MSH2, and MSH6 was observed in pMMR EC (lower panel) (original magnification, 40×). ADC, Apparent diffusion coefficient; d, cell diameter; ${D}_{{ex}}$, Extracellular diffusivity; dMMR, Mismatch repair deficient; EC, Endometrial cancer; H&E, Hematoxylin and eosin; IHC, Immunohistochemistry; PGSE, Pulsed gradient spin echo; pMMR, Mismatch repair proficient; T1-enhanced, Contrast-enhanced T1-weighted imaging; T2WI, T2-weighted imaging; ${v}_{{in}}$ Intracellular volume fraction; 20 Hz, Oscillating gradient spin echo at 20 Hz; 40 Hz, Oscillating gradient spin echo at 40 Hz; ρ Cellularity

One hundred and thirty-one consecutive patients with suspicious endometrial pathology underwent MRI with diffusion-weighted MRI acquired using PGSE and OGSE sequences. After exclusion, 82 patients (aged 59.3 ± 11.4 years, mean ± standard deviation) were included in the final study cohort (Fig. 1). There were 17 dMMR EC patients (20.7%) and 65 pMMR EC patients (79.3%). The baseline characteristics of the patients are summarized in Table 2. The microstructural feature maps and MRI images of two representative MMR tumors are presented in Fig. 2.

**Table 2:** Clinicopathological characteristics of the patients

Characteristic		dMMR EC (*n* = 17)	pMMR EC (*n* = 65)	*p*
Age (years)		61.8 ± 10.1	58.6 ± 11.8	0.28^a^
BMI (kg/m^2^)		24.0 (5.46)	23.6 (5.55)	0.73^b^
Menopause	Yes	14 (82.4)	52 (80.0)	> 0.9999^c^
	No	3 (17.6)	13 (20.0)	
FIGO stage	I	8 (47.1)	35 (53.8)	0.97^c^
	II	3 (17.6)	10 (15.4)	
	III	5 (29.4)	17 (26.2)	
	IV	1 (5.9)	3 (4.6)	
Grade	Low-grade	14 (82.4)	42 (64.6)	0.24^c^
	High-grade	3 (17.6)	23 (35.4)	
Histology	Endometrioid-EC	15 (88.2)	57 (87.7)	> 0.9999^c^
	Non-endometrioid-EC	2 (11.8)	8 (12.3)	
DMI	Positive	9 (52.9)	30 (46.2)	0.79^c^
	Negative	8 (47.1)	35 (53.8)	
LVSI	Positive	6 (35.3)	16 (24.6)	0.37^c^
	Negative	11 (64.7)	49 (75.4)	
CSI	Positive	4 (23.5)	14 (21.5)	> 0.9999^c^
	Negative	13 (76.5)	51 (78.5)	
LNM	Positive	5 (29.4)	18 (27.7)	> 0.9999^c^
	Negative	12 (70.6)	47 (72.3)	
ER	Positive	16 (94.1)	58 (89.2)	> 0.9999^c^
	Negative	1 (5.9)	7 (10.8)	
PR	Positive	15 (88.2)	49 (75.4)	0.34^c^
	Negative	2 (11.8)	16 (24.6)	

Data are presented as mean ± standard deviation, median with interquartile range, or patient number with percentage.

*BMI* Body mass index, *CSI* Cervical stromal invasion, *DMI* Deep myometrial invasion, *dMMR* Mismatch repair deficient, *EC* Endometrial cancer, *ER* Estrogen receptor, *LNM* Lymph node metastasis, *LVSI* Lymphovascular space invasion, *pMMR* Mismatch repair proficient, *PR* Progesterone receptor

^a^ Comparisons were performed by Welch’s *t*-test

^b^ Comparisons were performed by Mann–Whitney *U*-test

^c^ Comparisons were performed by Fisher’s test

### Correlation with histopathological findings

**Fig. 3:**
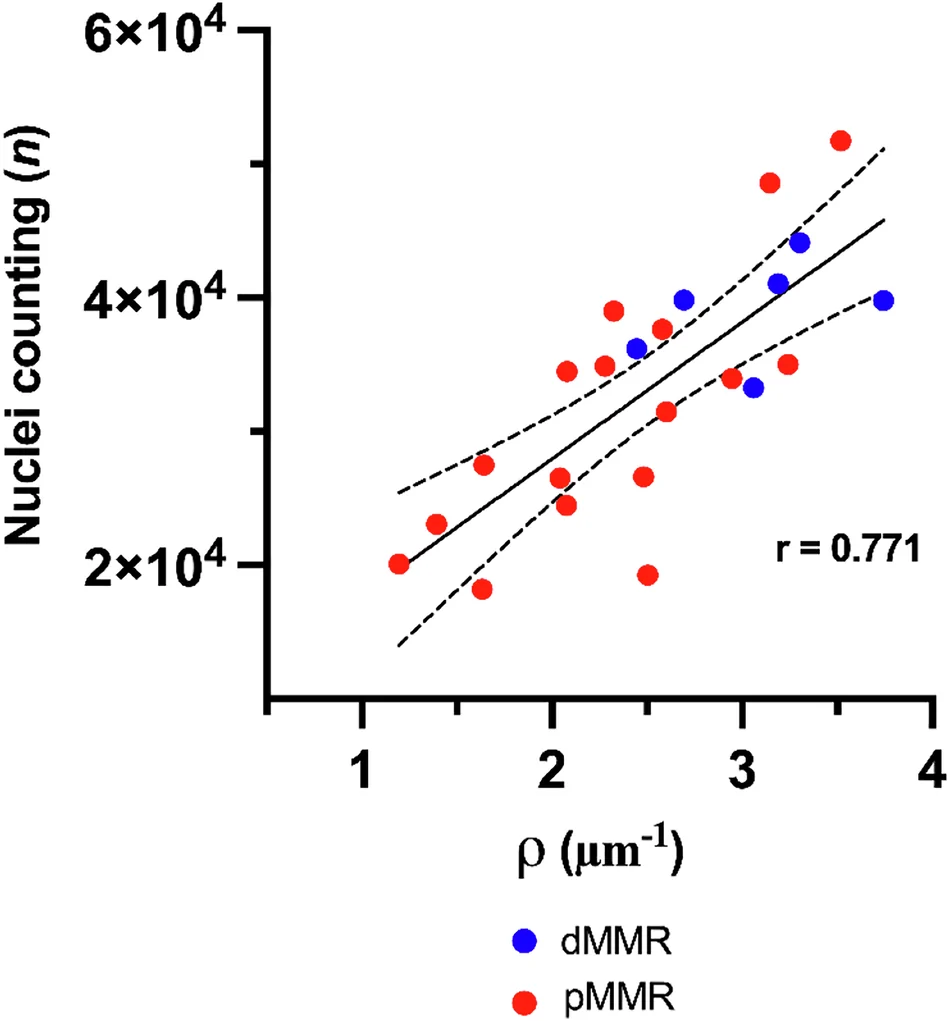
Correlation between td-dMRI-derived cell density (ρ) estimated from the IMPULSED method and nuclei counting from hematoxylin and eosin staining. dMMR, Mismatch repair deficient; EC, Endometrial cancer; pMMR, Mismatch repair proficient; td-dMRI, Time-dependent diffusion magnetic resonance imaging; ρ, Cellularity

There was a positive correlation between histological cell density and td-dMRI-derived cell density (${{\rm{\rho }}}$) estimated from the IMPULSED methods (*r* = 0.771, *p* < 0.001; 23 patients, dMMR EC, *n* = 6, pMMR EC, *n* = 17) (Fig. 3).

### Imaging metrics in different MMR status

**Fig. 4:**
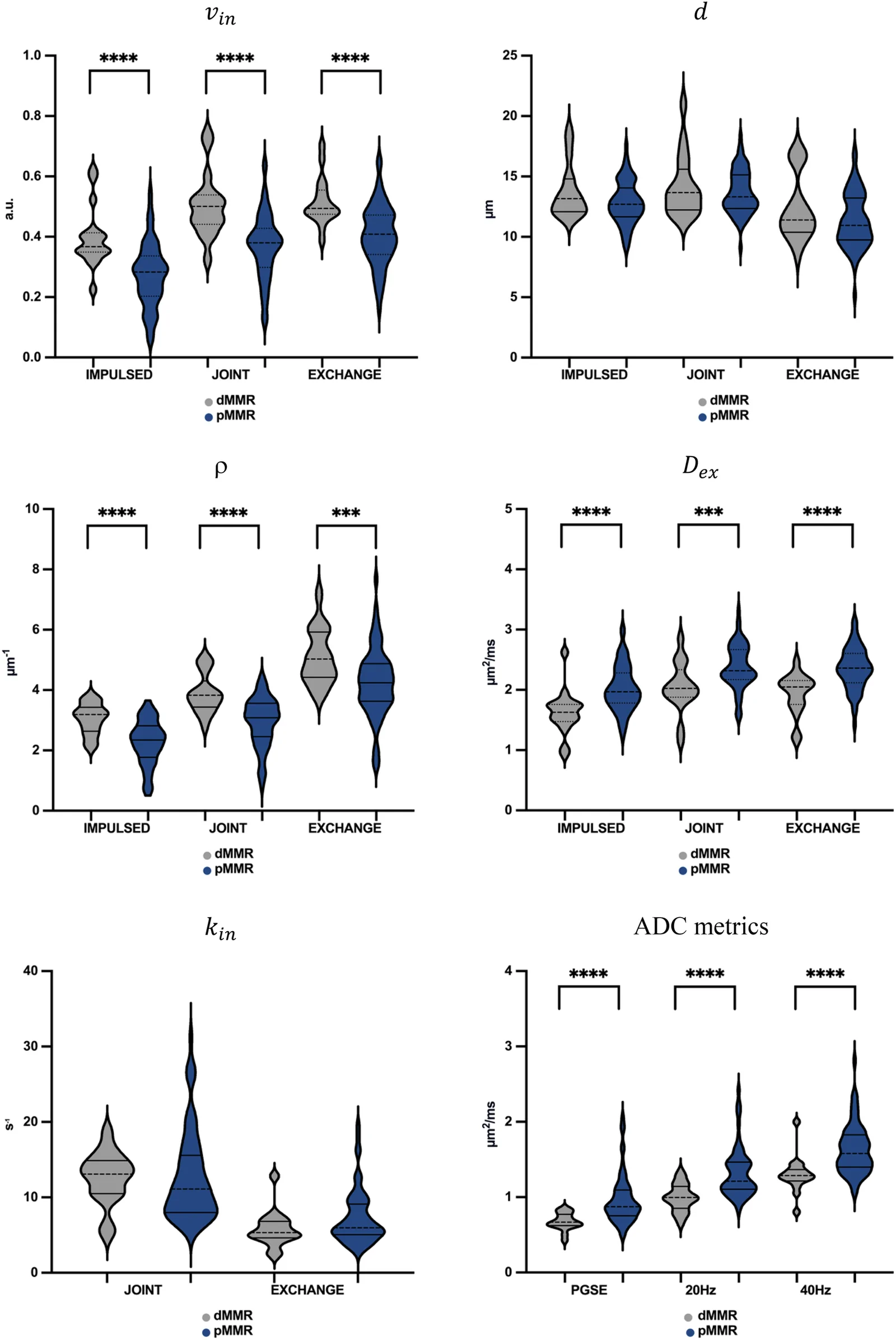
Intergroup comparison of microstructural parameters between dMMR EC and pMMR EC. Note: *p < 0.05; **p < 0.01; ***p < 0.001; ****p < 0.0001. ADC, Apparent diffusion coefficient; d, Cell diameter; ${D}_{{ex}}$, Extracellular diffusivity; dMMR, Mismatch repair deficient; EC, Endometrial cancer; ${k}_{{in}}$, Water exchange rate constant; PGSE, Pulsed gradient spin echo; pMMR, Mismatch repair proficient; ${v}_{{in}}$, Intracellular volume fraction; 20 Hz, Oscillating gradient spin echo at 20 Hz; 40 Hz, Oscillating gradient spin echo at 40 Hz; ρ, Cellularity

Inter-reader ICCs ranged from 0.852 to 0.982, and intra-reader ICCs ranged from 0.963 to 0.990, indicating a good to excellent reliability for all parameters. The microstructural parameters derived from the three methods (IMPULSED, JOINT, and EXCHANGE) demonstrated concordant trends in dMMR and pMMR EC (Table 3 and Fig. 4). The dMMR EC had significantly higher ${v}_{{in}}$ than the pMMR EC. The difference in cellularity ${{\rm{\rho }}}$ derived from all three methods was significantly different between the two MMR statuses, with ${{\rm{\rho }}}$ being higher in the dMMR group. On the contrary, ${D}_{{ex}}$ was significantly lower in dMMR than pMMR EC for all three methods. No significant difference was observed in cell diameter d for all three methods and in ${k}_{{in}}$ derived from JOINT and EXCHANGE. The ADC metrics measured by diffusion-weighted MRI were significantly different between the two groups, being lower in dMMR tumors.

**Table 3:** Intergroup comparison of microstructural parameters and ADC metrics between dMMR ECs and pMMR ECs

Method	Parameter	dMMR EC	pMMR EC	*p*
IMPULSED	${v}_{{in}}$ (a.u.)	0.367 (0.056)	0.283 (0.125)	**< 0.0001**
	d (μm)	13.2 (2.51)	12.7 (2.35)	0.241
	${{\rm{\rho }}}$ (μm^-1^)	3.19 (0.609)	2.34 (0.977)	**< 0.0001**
	${D}_{{ex}}$ (μm^2^/ms)	1.63 (0.251)	1.97 (0.476)	**< 0.0001**
JOINT	${v}_{{in}}$ (a.u.)	0.500 (0.088)	0.380 (0.124)	**< 0.0001**
	d (μm)	13.7 (2.81)	13.3 (2.76)	0.767
	${{\rm{\rho }}}$ (μm^-1^)	3.83 (0.464)	3.08 (1.09)	**< 0.0001**
	${D}_{{ex}}$ (μm^2^/ms)	2.03 (0.337)	2.32 (0.478)	**0.001**
	${k}_{{in}}$ (s^-1^)	13.1 (3.86)	11.1 (7.23)	0.401
EXCHANGE	${v}_{{in}}$ (a.u.)	0.494 (0.067)	0.409 (0.128)	**< 0.0001**
	d (μm)	11.4 (2.36)	11.0 (3.39)	0.194
	${{\rm{\rho }}}$ (μm^-1^)	5.03 (1.40)	4.24 (1.23)	**0.003**
	${D}_{{ex}}$ (μm^2^/ms)	2.05 (0.376)	2.36 (0.479)	**< 0.0001**
	${k}_{{in}}$ (s^-1^)	5.33 (2.09)	5.97 (3.92)	0.112
ADC metrics	ADC_PGSE_ (μm^2^/ms)	0.669 (0.131)	0.873 (0.329)	**< 0.0001**
	ADC_20Hz_ (μm^2^/ms)	0.996 (0.244)	1.21 (0.355)	**< 0.0001**
	ADC_40Hz_ (μm^2^/ms)	1.29 (0.127)	1.58 (0.410)	**< 0.0001**

Data are presented as median with interquartile range. Bold font indicates statistical significance

*ADC* Apparent diffusion coefficient, d Cell diameter, ${D}_{{ex}}$ Extracellular diffusivity, *dMMR* Mismatch repair deficient, *EC* Endometrial cancer, ${k}_{{in}}$ Water exchange rate constant, *PGSE* Pulsed gradient spin echo, *pMMR* Mismatch repair proficient, ${v}_{{in}}$ Intracellular volume fraction, *20 Hz* Oscillating gradient spin echo at 20Hz, *40 Hz* Oscillating gradient spin echo at 40 Hz, ρ Cellularity

### Diagnostic performance of imaging metrics

As illustrated in Table 4, using the IMPULSED method, ${v}_{{in}}$ provided the best discrimination with an AUC of 0.831. For the JOINT method, ${v}_{{in}}$ exhibited the highest AUC of 0.838, followed closely by ${{\rm{\rho }}}$ with an AUC of 0.821; whereas using the EXCHANGE method, ${v}_{{in}}$ and ${D}_{{ex}}$ were the top two discriminators, with AUCs of 0.824 and 0.815, respectively. All single ADCs showed similar diagnostic performances. Combining the microstructural parameters improved the diagnostic performance over ADC alone: IMPULSED, the combination of ${v}_{{in}}$ and ${D}_{{ex}}$ enhanced the AUC to 0.908 (DeLong test: *p* = 0.025); JOINT methods, the combination of ${v}_{{in}}$ and ${D}_{{ex}}$ improved the AUCs to 0.903 (DeLong test: *p* = 0.046); for EXCHANGE, the combination of ${v}_{{in}}$ and ${D}_{{ex}}$ improved the AUCs to 0.876 without significant difference (DeLong test: *p* = 0.325). Bootstrap internal validation demonstrated limited optimism across all three combination models (optimism = 0.012–0.013) (Supplementary Table S2). Sensitivity analysis demonstrated that scanner type was not a significant predictor in any of the three models (all *p* > 0.640). When evaluated by leave-one-out cross-validation–LOOCV, inclusion of scanner type consistently decreased model performance (Supplementary Table S3).

**Table 4:** Diagnostic performance of ADC metrics and microstructural parameters derived from three methods in differentiating dMMR ECs and pMMR ECs

Method	Parameter	AUC (95% CI)	Accuracy (%)	Sensitivity (%)	Specificity (%)	PPV (%)	NPV (%)
IMPULSED	${v}_{{in}}$	0.831 (0.734, 0.928)	74.4 (61/82)	94.1 (16/17)	69.2 (45/65)	44.4 (16/36)	97.8 (45/46)
	d	0.594 (0.448,0.740)	41.5 (34/82)	94.1 (16/17)	27.7 (18/65)	25.4 (16/63)	94.7 (18/19)
	${{\rm{\rho }}}$	0.808 (0.700, 0.917)	76.8 (63/82)	70.6 (12/17)	78.5 (51/65)	46.2 (12/26)	91.1 (51/56)
	${D}_{{ex}}$	0.809 (0.690, 0.928)	79.3 (65/82)	82.6 (14/17)	78.5 (51/65)	50.0 (14/28)	94.0 (51/54)
	(${v}_{{in}}$, ${D}_{{ex}}$)	0.908 (0.842, 0.974)	86.6 (72/82)	94.1 (16/17)	84.6 (55/65)	61.5 (16/26)	98.2 (55/56)
JOINT	${v}_{{in}}$	0.838 (0.738, 0.938)	73.2 (60/82)	94.1 (16/17)	67.7 (44/65)	43.5 (16/37)	97.8 (44/45)
	d	0.524 (0.363, 0.685)	79.3 (65/82)	17.6 (3/17)	95.4 (62/65)	50.0 (3/6)	81.4 (62/76)
	${{\rm{\rho }}}$	0.821 (0.721, 0.921)	67.1 (55/82)	94.1 (16/17)	60.0 (39/65)	38.5 (16/42)	97.5 (39/40)
	${D}_{{ex}}$	0.753 (0.613, 0.893)	80.5 (66/82)	70.6 (12/17)	83.1 (54/65)	52.2 (12/23)	91.1 (54/59)
	${k}_{{in}}$	0.567 (0.426, 0.709)	53.7 (44/82)	88.2 (15/17)	44.6 (29/65)	29.5 (15/51)	93.0 (29/31)
	(${v}_{{in}}$, ${D}_{{ex}}$)	0.903 (0.838, 0.969)	86.6 (71/82)	94.1 (16/17)	84.6 (55/65)	61.5 (16/26)	98.2 (55/56)
EXCHANGE	${v}_{{in}}$	0.824 (0.730, 0.918)	75.6 (62/82)	88.2 (15/17)	72.3 (47/65)	45.9 (15/33)	95.2 (47/49)
	d	0.604 (0.458, 0.749)	45.1 (37/82)	94.1 (16/17)	32.3 (21/65)	26.7 (16/60)	95.4 (21/22)
	${{\rm{\rho }}}$	0.731 (0.613, 0.850)	61.0 (50/82)	82.6 (14/17)	55.4 (36/65)	32.5 (14/43)	92.0 (36/39)
	${D}_{{ex}}$	0.815 (0.716, 0.915)	76.8 (63/82)	88.2 (15/17)	73.8 (48/65)	46.8 (15/32)	96.0 (48/50)
	${k}_{{in}}$	0.626 (0.486, 0.766)	46.3 (38/82)	94.1 (16/17)	33.8 (22/65)	27.1 (16/59)	95.5 (22/23)
	(${v}_{{in}}$, ${D}_{{ex}}$)	0.876 (0.803, 0.949)	81.7 (67/82)	94.1 (16/17)	78.5 (51/65)	53.3 (16/30)	98.1 (51/52)
ADC metrics	ADC_PGSE_	0.839 (0.751, 0.927)	69.5 (57/82)	100 (17/17)	61.5 (40/65)	40.0 (17/42)	100 (40/40)
	ADC_20Hz_	0.832 (0.728, 0.935)	84.1 (69/82)	70.6 (12/17)	87.7 (57/65)	60.0 (12/20)	91.5 (57/62)
	ADC_40Hz_	0.834 (0.723, 0.944)	73.2 (60/82)	94.1 (16/17)	67.7 (44/65)	43.5 (16/37)	97.8 (44/45)

Accuracy, sensitivity, specificity, PPV, and NPV are presented as percentages (number of participants)

*ADC* Apparent diffusion coefficient, *AUC* Area under the receiver-operating characteristic curve, *CI* Confidence interval, d Cell diameter, ${D}_{{ex}}$ Extracellular diffusivity, *dMMR* Mismatch repair deficient, *EC* Endometrial cancer, ${k}_{{in}}$ Water exchange rate constant, *NPV* Negative predictive value *PGSE* Pulsed gradient spin echo, *PPV* Positive predictive value, *pMMR* Mismatch repair proficient, ${v}_{{in}}$ Intracellular volume fraction, *20 Hz* Oscillating gradient spin echo at 20Hz, *40 Hz* Oscillating gradient spin echo at 40 Hz, ρ Cellularity

### Comparison of combination models across three methods

Among the three combination models, EXCHANGE was excluded as it failed to significantly outperform ADC_PGSE_, while IMPULSED and JOINT showed equivalent discrimination without significant difference. Bootstrap internal validation confirmed robust performance for both, with JOINT showing marginally better calibration in calibration slope and Hosmer–Lemeshow test (Supplementary Table S2). For ${D}_{{ex}}$, JOINT achieved a lower standard error of ln(OR) than IMPULSED (0.402 *versus* 0.466), indicating superior parametric precision, while ${v}_{{in}}$ precision was comparable (0.471 *versus* 0.461) (Supplementary Table S4). Therefore, JOINT was selected as the preferred combination model.

## Discussion

In this study, microstructural parameters obtained from td-dMRI were different between dMMR and pMMR EC, showing higher ${v}_{{in}}$, ${{\rm{\rho }}}$ and lower ${D}_{{ex}}$, ADC values in dMMR EC, consistently observed using IMPULSED, JOINT, and EXCHANGE methods. The combination of these parameters significantly improved the diagnostic efficacy in distinguishing the two molecular subtypes of EC, demonstrating the potential role of td-dMRI in distinguishing MMR status of EC, providing a noninvasive approach for therapeutic guidance.

The treatment landscape of EC is evolving based on the molecular classification. The dMMR EC made up 20.7% (17/82) of the study cohort, comparable to 20–30% prevalence reported in the literature [6]. Determination of MMR status is recommended not only for screening for Lynch syndrome but also to promote prognostic assessment and therapeutic stratification in EC [32–34]. PD-1/PD-L1 targeted immunotherapy has demonstrated clinical efficacy in EC, especially in tumors with high microsatellite instability or dMMR [35]. KEYNOTE-158 study identified tumors with dMMR benefitting from Pembrolizumab [36]. This result was further supported by the GARNET study, which showed durable anti-tumor activity of Dostarlimab in this subtype [37].

The reference standard to determine MMR status is IHC testing, which depends on the pathological examination of biopsy or surgical specimens. Although IHC testing is widely available, it is labor-intensive with a multistep protocol including preparation and manual analysis [38]. Moreover, the pathological assessment is affected by tumor heterogeneity, sampling error, and small biopsy specimens. The dependence on tissue availability requires coordinated scheduling between the surgery and pathological assessment, often resulting in delay in ascertaining the MMR status. On the other hand, MRI examination is an integral part of the preoperative assessment with the ability to predict important histopathological factors of EC, like myometrial invasion and cervical stromal involvement, in addition to nodal staging [39, 40]. The diffusion-weighted MRI-derived ADC metrics were used to differentiate MMR status in previous studies, with preliminary trends observed, but the clinical performance was limited [41, 42]. The emergence of td-dMRI may provide new insights in addressing this clinical gap in knowledge. More notably, it provides a preoperative evaluation, giving a time-sensitive advantage over IHC.

The biological interpretations combine two elements: quantitative differences observed in our cohort, and mechanistic inferences drawn from prior literature. The latter, particularly those involving immune infiltration, extracellular matrix remodeling, and tumor hypoxia, were not directly tested in this study and should be regarded as hypothesis-generating. The significantly elevated ${v}_{{in}}$ and ${{\rm{\rho }}}$ in dMMR EC may be related to the impaired DNA repair and genomic instability of dMMR tumors that have been reported to increase neoantigen load on the surface of tumor cells, rendering them more prone to attack by lymphocytes and other immune cells [43]. It has previously been observed that the expression levels of T cells, macrophages, and NK cells in dMMR tumors were significantly higher than in pMMR tumors, which could potentially further account for a higher cell density in dMMR EC, consistent with the trend observed in our study [44]. The increased ${{\rm{\rho }}}$ in dMMR tumors would in turn compress the extracellular space, which was consistent with the lower ${D}_{{ex}}$ observed in this group. Additionally, remodeling of the extracellular matrix and altered angiogenic activities in dMMR tumors may also lead to restricted extracellular diffusion, even if the extracellular space is not compressed [45, 46]. However, these specific cellular and matrix mechanisms were not directly assessed in the present study and remain to be confirmed by dedicated histological or IHC analyses. Of note, the extracellular space in the current multi-compartment methods included the entire space outside the cells, encompassing the intravascular space, and therefore ${D}_{{ex}}$ should be interpreted as reflecting the combined effect of these extracellular changes.

It is important to note that the microstructural differences observed here may reflect biological features of the dMMR molecular subtype that are not fully captured by conventional descriptors of tumor aggressiveness such as grade, stage, or depth of invasion. In our cohort, conventional clinicopathological features did not differ significantly between dMMR and pMMR groups (Table 2). However, given the limited number of dMMR cases (*n* = 17), these comparisons were underpowered, and we cannot exclude clinically meaningful imbalances.

A further line of evidence supporting the biological plausibility of MRI-based MMR prediction comes from the emerging connection between MMR deficiency and tumor hypoxia. Although the most direct evidence to date has been generated in colorectal cancer, where dMMR tumors have been reported to exhibit distinctive hypoxic and metabolic microenvironmental features [47], the underlying mechanism is not organ-specific and is therefore plausibly relevant to EC as well. In parallel, multiple quantitative MRI metrics have been shown to be predictive of intratumoral hypoxia in other tumor types [48–50], supporting the broader concept of MRI as a noninvasive surrogate for the hypoxic microenvironment. Taken together, these observations suggest a coherent biological chain, in which dMMR status leads to an altered hypoxic and microvascular microenvironment, in turn, to MRI-detectable microstructural and perfusion changes. That is consistent with the discriminative performance of our td-dMRI parameters. This framework also resonates with the microvascular dimension of our findings, where ${D}_{{ex}}$ differed significantly between dMMR and pMMR tumors, and provides a mechanistic rationale linking the molecular phenotype of MMR deficiency to its imaging signature. We acknowledge that direct evidence for the dMMR–hypoxia–imaging axis in EC is currently lacking, and dedicated studies in this disease setting are warranted to confirm and extend the cross-tumor observations cited above.

We also systematically compared the performance of the three methods, particularly between the most commonly used IMPULSED, which maintains the classic impermeable sphere assumption, and the recently emerging JOINT and EXCHANGE methods that incorporate transcytolemmal water exchange. Among the three combination models, EXCHANGE failed to significantly outperform ADC_PGSE_, while IMPULSED and JOINT achieved comparable discriminative performance with no statistically significant difference. Nonetheless, the three models diverged substantially in parameter-level behavior, and JOINT was ultimately selected as the preferred method based on its superior parametric precision for ${D}_{{ex}}$ and marginally better calibration. The retained predictors ${v}_{{in}}$ and ${D}_{{ex}}$ further provide a biologically complementary two-compartment fingerprint, reflecting intracellular cellularity and extracellular stromal architecture, respectively.

Despite this parametric advantage, incorporating transcytolemmal water exchange did not improve MMR status discrimination, which is somewhat unexpected given prior evidence that JOINT and EXCHANGE can extract more accurate microstructural parameters than IMPULSED [28, 29], and that exchange-sensitive modeling aids breast tumor characterization [51]. This likely reflects the fundamentally limited sensitivity to transcytolemmal exchange at the OGSE frequencies (20 and 40 Hz) achievable on current clinical scanners, where effective diffusion times remain considerably shorter than the intracellular water residence time. Furthermore, the estimated ${k}_{{in}}$ may be confounded by perfusion and intracellular–intravascular water exchange, introducing systematic bias that further attenuates its discriminative sensitivity. Although td-dMRI faces challenges in clinical implementation related to specialized acquisition protocols, preprocessing steps, and dedicated analysis software, recent advances in hardware and open-source platforms have substantially lowered these barriers. In our workflow, the joint OGSE–PGSE protocol is available on a commercial sharing platform and can be acquired in approximately 8 min on clinically available 3.0-T scanners, with preprocessing and biophysical model fitting readily executable on a standard personal computer using the open-source MATI software package (https://github.com/jzxu0622/mati). These developments substantially enhance the clinical feasibility of this advanced imaging technique.

The current study had several limitations. First, the 20 mm² lesion size threshold, constrained by the spatial resolution of DWI, may bias the cohort toward larger tumors. Extending td-dMRI to sub-centimeter EC lesions through higher-resolution diffusion acquisitions, advanced denoising techniques, and small-lesion-adapted modeling strategies represents an important direction for future work. Second, the relatively small sample size, class imbalance, and slightly low events per variable warrant cautious interpretation. External validation in an independent cohort would be necessary before clinical implementation. The sensitivity analysis confirmed that inter-scanner differences did not confound the results. Nevertheless, both scanners were from a single vendor with similar acquisition protocols.

In addition, this was a single-center study in which all participants were preoperatively recruited patients with suspected EC, which may limit generalizability with respect to histological subtype distribution and demographic characteristics, and introduces an inherent selection bias. Prospective multicenter, multi-vendor validation in more heterogeneous cohorts therefore remains important to test the robustness and clinical transportability of the results. Third, manual tumor delineation is both time-consuming and labor-intensive, and results are reader-dependent. Implementing a high-precision, automatic segmentation system would streamline the process. Fourth, modeling cells as ideal spheres, which is the most common approximation in tumor imaging, represents a fundamental limitation of current quantitative models, as *in vivo* tissues comprise multiple cell populations with heterogeneous sizes and non-spherical morphologies, such that the derived cell diameter d reflects only an equivalent size corresponding to the average cell volume. Future corrections will primarily rely on advances in theoretical modeling, such as constructing more comprehensive frameworks incorporating multiple cell populations or developing correction formulas to account for deviations from spherical geometry. Fifth, the current statistical analysis of imaging results was implemented on a VOI-wise or patient-wise basis, *i.e.*, only using the mean values of microstructural parameters and ADC metrics to construct logistic regression models. This simplified analysis strategy did not account for intra-tumor heterogeneity. More advanced methods, such as radiomics and habitat analysis, could capture this heterogeneous information and further improve the diagnostic efficacy of td-dMRI. Finally, the histopathological correlation was restricted to 23 of 82 patients, as whole-slide digitization was not routine and was performed only for slides meeting digital-analysis quality requirements. This modest subset weakens the biological validation, and prospective whole-slide imaging in larger cohorts is needed for definitive confirmation.

## Conclusion

In conclusion, in this exploratory study, td-dMRI was capable of discriminating dMMR and pMMR EC, providing information on the unique microstructural organization in these tumors. The derived microstructural parameters (${v}_{{in}}$ and ${D}_{{ex}}$) could potentially serve as a useful method to improve the diagnostic performance of diffusion-weighted MRI in determining the MMR status of EC, but further external validation is required.

## Supplementary information

**Additional file 1: Table S1.** Parameters of routine sequences. **Table S2.** Bootstrap Internal validation and calibration assessment of multivariate logistic regression models for predicting MMR status. **Table S3.** Sensitivity analysis in multivariate logistic regression models. **Table S4.** Comparison of combination models across three td-dMRI methods. **Fig. S1** Discretization of arbitrary waveform.

## Data Availability

The datasets used and/or analyzed during the current study are available from the corresponding author on reasonable request.

## Abbreviations

AUC: Area under the receiver-operating characteristic curve
ADC: Apparent diffusion coefficient
d: Cell diameter
$${D}_{{ex}}$$: Extracellular diffusivity
dMMR: Mismatch repair deficient
DWI: Diffusion-weighted imaging
EC: Endometrial cancer
ICC: Intraclass correlation coefficient
IHC: Immunohistochemistry
${k}_{{in}}$: Water exchange rate constant
MMR: Mismatch repair
MRI: Magnetic resonance imaging
OGSE: Oscillating gradient spin-echo
PD-L1: Programmed death-ligand 1
PGSE: Pulsed gradient spin-echo
pMMR: Mismatch repair proficient
td-dMRI: Time-dependent diffusion MRI
${v}_{{in}}$: Intracellular volume fraction
VOI: Volume of interest
ρ: Cellularity

## References

[1] Anca-Stanciu MB, Manu A, Olinca MV et al (2025) Comprehensive review of endometrial cancer: new molecular and FIGO classification and recent treatment changes. J Clin Med 14:1385. 10.3390/jcm14041385 PMC11856752 40004914

[2] Concin N, Matias-Guiu X, Vergote I et al (2021) ESGO/ESTRO/ESP guidelines for the management of patients with endometrial carcinoma. Radiother Oncol 154:327–353. 10.1016/j.radonc.2020.11.018 33712263

[3] Kandoth C, Schultz N, Cherniack AD et al (2013) Integrated genomic characterization of endometrial carcinoma. Nature 497:67–73. 10.1038/nature12113 PMC3704730 23636398

[4] van den Heerik A, Horeweg N, de Boer SM et al (2021) Adjuvant therapy for endometrial cancer in the era of molecular classification: radiotherapy, chemoradiation and novel targets for therapy. Int J Gynecol Cancer 31:594–604. 10.1136/ijgc-2020-001822 PMC8020082 33082238

[5] Lee JHS, Li JJX, Chow C et al (2021) Long-term survival and clinicopathological implications of DNA mismatch repair status in endometrioid endometrial cancers in Hong Kong Chinese women. Biomedicines. 10.3390/biomedicines9101385 PMC8533409 34680502

[6] Mendiola M, Heredia-Soto V, Ruz-Caracuel I et al (2023) Comparison of methods for testing mismatch repair status in endometrial cancer. Int J Mol Sci 24:14468. 10.3390/ijms241914468 PMC10572657 37833916

[7] Addante F, d’Amati A, Santoro A et al (2024) Mismatch repair deficiency as a predictive and prognostic biomarker in endometrial cancer: a review on immunohistochemistry staining patterns and clinical implications. Int J Mol Sci 25:1056. 10.3390/ijms25021056 PMC10816607 38256131

[8] Li JJX, Ip PPC (2022) Endometrial cancer: An update on prognostic pathologic features and clinically relevant biomarkers. Surg Pathol Clin 15:277–299. 10.1016/j.path.2022.02.006 35715162

[9] O’Malley DM, Bariani GM, Cassier PA et al (2022) Pembrolizumab in patients with microsatellite instability-high advanced endometrial cancer: results from the KEYNOTE-158 study. J Clin Oncol 40:752–761. 10.1200/jco.21.01874 PMC8887941 34990208

[10] Loukola A, Salovaara R, Kristo P et al (1999) Microsatellite instability in adenomas as a marker for hereditary nonpolyposis colorectal cancer. Am J Pathol 155:1849–1853. 10.1016/s0002-9440(10)65503-4 PMC1866944 10595914

[11] Di Dio C, Bogani G, Di Donato V et al (2023) The role of immunotherapy in advanced and recurrent mmr deficient and proficient endometrial carcinoma. Gynecol Oncol 169:27–33. 10.1016/j.ygyno.2022.11.031 36493574

[12] Antill Y, Kok PS, Robledo K et al (2021) Clinical activity of durvalumab for patients with advanced mismatch repair-deficient and repair-proficient endometrial cancer. A nonrandomized phase 2 clinical trial. J Immunother Cancer 9:e002255. 10.1136/jitc-2020-002255 PMC8190057 34103352

[13] Oaknin A, Gilbert L, Tinker AV et al (2022) Safety and antitumor activity of dostarlimab in patients with advanced or recurrent DNA mismatch repair deficient/microsatellite instability-high (dMMR/MSI-H) or proficient/stable (MMRp/MSS) endometrial cancer: interim results from GARNET-a phase I, single-arm study. J Immunother Cancer 10:e003777. 10.1136/jitc-2021-003777 PMC8785197 35064011

[14] Liu C, Ping H, Yao M et al (2025) Traditional and new views on MSI-H/dMMR endometrial cancer. Biomolecules 15:1370. 10.3390/biom15101370 PMC12562029 41154599

[15] Casas-Arozamena C, Moiola CP, Vilar A et al (2023) Noninvasive detection of microsatellite instability in patients with endometrial cancer. Int J Cancer 152:2206–2217. 10.1002/ijc.34435 36650670

[16] Bodalal Z, Hong EK, Trebeschi S et al (2024) Non-invasive CT radiomic biomarkers predict microsatellite stability status in colorectal cancer: a multicenter validation study. Eur Radiol Exp 8:98. 10.1186/s41747-024-00484-8 PMC11347521 39186200

[17] Mallardi D, Danti G, Galluzzo A et al (2025) Radiomics-based prediction of microsatellite instability in colorectal cancer: a non-invasive approach to treatment stratification. Radiol Med 130:1731–1741. 10.1007/s11547-025-02081-0 40892331

[18] Jia Y, Hou L, Zhao J et al (2024) Radiomics analysis of multiparametric MRI for preoperative prediction of microsatellite instability status in endometrial cancer: a dual-center study. Front Oncol 14:1333020. 10.3389/fonc.2024.1333020 PMC10860747 38347846

[19] Wang Y, Xie B, Wang K et al (2025) Multi-parametric MRI habitat radiomics based on interpretable machine learning for preoperative assessment of microsatellite instability in rectal cancer. Acad Radiol 32:3975–3988. 10.1016/j.acra.2025.02.009 40016002

[20] Gil RT, Cunha TM, Horta M et al (2019) The added value of diffusion-weighted imaging in the preoperative assessment of endometrial cancer. Radiol Bras 52:229–236. 10.1590/0100-3984.2018.0054 PMC6696747 31435083

[21] Xu J (2021) Probing neural tissues at small scales: recent progress of oscillating gradient spin echo (OGSE) neuroimaging in humans. J Neurosci Methods 349:109024. 10.1016/j.jneumeth.2020.109024 PMC10124150 33333089

[22] Wu D, Jiang K, Li H et al (2022) Time-dependent diffusion MRI for quantitative microstructural mapping of prostate cancer. Radiology 303:578–587. 10.1148/radiol.211180 35258368

[23] Wang X, Zhang Y, Cheng J et al (2025) Microstructural diffusion MRI for differentiation of breast tumors and prediction of prognostic factors in breast cancer. Front Oncol 15:1498691. 10.3389/fonc.2025.1498691 PMC11919649 40110196

[24] Ejima F, Fukukura Y, Kamimura K et al (2024) Oscillating gradient diffusion-weighted MRI for risk stratification of uterine endometrial cancer. J Magn Reson Imaging 60:67–77. 10.1002/jmri.29106 37886909

[25] Zhao Y, Zhao F, Cheng M et al (2025) Risk stratification prediction of endometrial cancer using microstructural mapping based on time-dependent diffusion MRI. Cancer Sci 116:1627–1637. 10.1111/cas.70036 PMC12127095 40059556

[26] Veraart J, Novikov DS, Christiaens D et al (2016) Denoising of diffusion MRI using random matrix theory. Neuroimage 142:394–406. 10.1016/j.neuroimage.2016.08.016 PMC5159209 27523449

[27] Jiang X, Li H, Xie J et al (2017) In vivo imaging of cancer cell size and cellularity using temporal diffusion spectroscopy. Magn Reson Med 78:156–164. 10.1002/mrm.26356 PMC5293685 27495144

[28] Jiang X, Devan SP, Xie J et al (2022) Improving mr cell size imaging by inclusion of transcytolemmal water exchange. NMR Biomed 35:e4799. 10.1002/nbm.4799 PMC10124991 35794795

[29] Shi D, Wang X, Li S et al (2025) Comprehensive characterization of tumor therapeutic response via simultaneous mapping of cell size, density, and transcytolemmal water exchange. Magn Reson Imaging 122:110433. 10.1016/j.mri.2025.110433 PMC12805662 40460946

[30] Xu J, Devan SP, Shi D et al (2025) MATI: a GPU-accelerated toolbox for microstructural diffusion MRI simulation and data fitting with a graphical user interface. Magn Reson Imaging 122:110428. 10.1016/j.mri.2025.110428 PMC12805661 40419173

[31] Xu J, Jiang X, Li H et al (2020) Magnetic resonance imaging of mean cell size in human breast tumors. Magn Reson Med 83:2002–2014. 10.1002/mrm.28056 PMC7047520 31765494

[32] Cho KR, Cooper K, Croce S et al (2019) International Society of Gynecologic Pathologists (ISGyP) endometrial cancer project: guidelines from the special techniques and ancillary studies group. Int J Gynecol Pathol 38:S114–s22. 10.1097/pgp.0000000000000496 PMC6296838 29521846

[33] Crosbie EJ, Ryan NAJ, Arends MJ et al (2019) The Manchester International Consensus Group recommendations for the management of gynecological cancers in lynch syndrome. Genet Med 21:2390–2400. 10.1038/s41436-019-0489-y PMC6774998 30918358

[34] Stinton C, Jordan M, Fraser H et al (2021) Testing strategies for lynch syndrome in people with endometrial cancer: systematic reviews and economic evaluation. Health Technol Assess 25:1–216. 10.3310/hta25420 PMC8273681 34169821

[35] Tommasi O, Marchetti M, Tripepi M et al (2025) PD-1 and PD-L1 expression in endometrial cancer: a systematic review of the literature. J Clin Med 14:401. 10.3390/jcm14020401 PMC11766114 39860407

[36] Marabelle A, Le DT, Ascierto PA et al (2020) Efficacy of pembrolizumab in patients with noncolorectal high microsatellite instability/mismatch repair-deficient cancer: results from the phase II KEYNOTE-158 study. J Clin Oncol 38:1–10. 10.1200/jco.19.02105 PMC8184060 31682550

[37] Oaknin A, Pothuri B, Gilbert L et al (2023) Safety, efficacy, and biomarker analyses of dostarlimab in patients with endometrial cancer: interim results of the phase I GARNET study. Clin Cancer Res 29:4564–4574. 10.1158/1078-0432.Ccr-22-3915 PMC10643997 37363992

[38] Das A, Tomita N, Syme KJ et al (2025) Cross-modality learning for predicting immunohistochemistry biomarkers from hematoxylin and eosin-stained whole slide images. Am J Pathol. 10.1016/j.ajpath.2025.08.014 PMC12799405 40946794

[39] Dane C, Bakir S (2019) The effect of myometrial invasion on prognostic factors and survival analysis in endometrial carcinoma. Afr Health Sci 19:3235–3241. 10.4314/ahs.v19i4.47 PMC7040328 32127901

[40] Ferriss JS, Brix W, Tambouret R et al (2010) Cervical stromal invasion predicting survival in endometrial cancer. Obstet Gynecol 116:1035–1041. 10.1097/AOG.0b013e3181f73fc2 20966686

[41] Li Y, Liu X, Wang X et al (2021) Using amide proton transfer-weighted MRI to non-invasively differentiate mismatch repair deficient and proficient tumors in endometrioid endometrial adenocarcinoma. Insights Imaging 12:182. 10.1186/s13244-021-01126-y PMC8665952 34894294

[42] Minamiguchi K, Takahama J, Uchiyama T et al (2018) Uterine endometrial carcinoma with DNA mismatch repair deficiency: magnetic resonance imaging findings and clinical features. Jpn J Radiol 36:429–436. 10.1007/s11604-018-0741-4 29728922

[43] Green AR, Aleskandarany MA, Ali R et al (2017) Clinical impact of tumor DNA repair expression and T-cell infiltration in breast cancers. Cancer Immunol Res 5:292–299. 10.1158/2326-6066.Cir-16-0195 28254786

[44] Narayanan S, Kawaguchi T, Peng X et al (2019) Tumor infiltrating lymphocytes and macrophages improve survival in microsatellite unstable colorectal cancer. Sci Rep 9:13455. 10.1038/s41598-019-49878-4 PMC6748965 31530839

[45] Su Z, He Y, You L et al (2024) SPP1+ macrophages and FAP+ fibroblasts promote the progression of pmmr gastric cancer. Sci Rep 14:26221. 10.1038/s41598-024-76298-w PMC11528032 39482333

[46] Yuan Z, Li Y, Zhang S et al (2023) Extracellular matrix remodeling in tumor progression and immune escape: From mechanisms to treatments. Mol Cancer 22:48. 10.1186/s12943-023-01744-8 PMC10007858 36906534

[47] Xu Y, Hu J, Cao C et al (2021) Distinct hypoxia-related gene profiling characterizes clinicopathological features and immune status of mismatch repair-deficient colon cancer. J Oncol 2021:2427427. 10.1155/2021/2427427 PMC8670907 34917146

[48] Bodalal Z, Bogveradze N, Ter Beek LC et al (2023) Radiomic signatures from T2W and DWI MRI are predictive of tumour hypoxia in colorectal liver metastases. Insights Imaging 14:133. 10.1186/s13244-023-01474-x PMC10361926 37477715

[49] McCabe A, Martin S, Rowe S et al (2024) Oxygen-enhanced MRI assessment of tumour hypoxia in head and neck cancer is feasible and well tolerated in the clinical setting. Eur Radiol Exp 8:27. 10.1186/s41747-024-00429-1 PMC10914657 38443722

[50] Wang D, Chen J, Ying Y et al (2024) Assessment of hypoxia and its dynamic evolution in glioblastoma via qbold MRI: a comparative study with metformin treatment. Eur Radiol Exp 8:134. 10.1186/s41747-024-00533-2 PMC11612089 39621211

[51] Liu F, Wu L, Luo X et al (2025) Evaluating the diagnostic performance of MR cytometry imaging in differentiating benign and malignant breast tumors. J Magn Reson Imaging. 10.1002/jmri.29757 40070029

