# Supplementary material for: Time-dependent diffusion MRI in determining mismatch repair status of endometrial cancer
Source: Eur Radiol Exp. 2026 Nov 3;10:167. doi: 10.1186/s41747-026-00830-y (PMC13731628; doi:10.1186/s41747-026-00830-y)
Supplement: Supplementary file 1 [file 41747_2026_830_MOESM1_ESM.pdf]

Time-dependent diffusion MRI in determining mismatch repair status of endometrial cancer
ELECTRONIC SUPPLEMENTARY MATERIAL

Supplementary Tables (S1-S4)

Table S1. Parameters of routine sequences.

	T1WI	T2WI	CE-T1WI
Sequence	Spin Echo	PROPELLER	VIBE
Plane	Axial	Axial	Axial
TR (msec)	633	3000	3.7
TE (msec)	8.3	105	1.2
FOV (cm)	24 × 36	23 × 23	24 × 28
Matrix Size	256 × 384	400 × 400	224 × 192
Slice Thickness (mm)	5.0	4.0	2.0

CE Contrast-enhanced, *FOV* Field of view, *TE* Echo time, *TR* Repetition time, *T1WI* T1-weighted imaging, *T2WI* T2-weighted imaging.

Table S2. Bootstrap Internal validation and calibration assessment of multivariate logistic regression models for predicting MMR status.

Method	Parameters	Apparent AUC	Optimism	Corrected AUC	Calibration Slope	Calibration Intercept	HL test p-value
IMPULSED	v_{in}, D_{ex}	0.908	0.013	0.895	0.882	-0.037	0.596
JOINT	v_{in}, D_{ex}	0.903	0.012	0.891	0.889	-0.042	0.651
EXCHANGE	v_{in}, D_{ex}	0.876	0.012	0.864	0.901	-0.037	0.307

AUC Area under the receiver operating characteristic curve, D_{ex} Extracellular diffusivity, *HL* Hosmer-Lemeshow, *MMR* Mismatch repair, v_{in} Intracellular volume fraction.

Table S3. Sensitivity analysis in multivariate logistic regression models.

Method	Parameters	LOOCV AUC (original)	LOOCV AUC (with scanner)	ΔAUC	Scanner p-value
IMPULSED	v_{in}, D_{ex}	0.878	0.869	-0.009	0.862
JOINT	v_{in}, D_{ex}	0.882	0.867	-0.015	0.837
EXCHANGE	v_{in}, D_{ex}	0.853	0.834	-0.019	0.640

AUC Area under the receiver operating characteristic curve, D_{ex} Extracellular diffusivity, *LOOCV* Leave-one-out cross-validation, *MMR* Mismatch repair.

Table S4. Comparison of combination models across three td-dMRI methods.

Methods	Predictor	OR (per 1SD)	95% CI	p	SE
IMPULSED	v_{in}	4.089	1.657-10.088	**0.002**	0.461
	D_{ex}	0.277	0.111-0.689	**0.006**	0.466
JOINT	v_{in}	4.594	1.824-11.571	**0.001**	0.471
	D_{ex}	0.369	0.168-0.811	**0.013**	0.402
EXCHANGE	v_{in}	2.915	1.239-6.861	**0.014**	/
	D_{ex}	0.363	0.165-0.801	**0.012**	/

Note: Bold font indicates statistical significance.

CI Confidence interval, D_{ex} Extracellular diffusivity; *SD* Standard deviation, *td-dMRI* Time- diffusion magnetic resonance imaging, v_{in} Intracellular volume fraction.

Appendix S1: Analytical expression of intracellular apparent restricted diffusion coefficient

IMPULSED

Diffusion-weighted signal assuming a Gaussian phase distribution can be written as:

$$S = S_0 \exp(-\phi) \tag{S1}$$

where S_0 is the non-diffusion-weighted signal and the echo attenuation factor ϕ can be expressed as the following form based on the velocity correlation function developed by Stepisnik:

$$\phi = \frac{\gamma^2}{2} \sum_k B_k \int_0^{\mathrm{TE}} dt_1 \int_0^{\mathrm{TE}} dt_2 \exp(-a_k D|t_1 - t_2|)\, g(t_1) g(t_2) \tag{S2}$$

where γ is the gyromagnetic ratio, TE is the echo time, $g(t)$ is the time-varying diffusion gradient, D is the intrinsic diffusion coefficient, B_k and a_k are microstructure-related coefficients, which have been obtained for special shaped boundary conditions such as cylinders and spheres. Based on the Eq. (S2), the analytical expression of restricted dMRI signals under diffusion sequences with arbitrary gradient waveform can be derived, both for the sine and cosine-modulated OGSE and traditional PGSE sequences.

For the PGSE with trapezoid-shaped gradient waveforms, the analytical expression of ϕ is given as:

$$\phi = \gamma^2 G^2 \sum_k \frac{B_k}{a_k^4 D^4 t_r^2} \left\{ \begin{array}{c} 2\exp(-a_k D t_p) - 4\exp(-a_k D t_r) - 4\exp(-a_k D \Delta) \\ +2\exp(-a_k D(\Delta - t_r)) + 2\exp(-a_k D(\Delta + t_r)) \\ -\exp\left(-a_k D(\Delta - t_p)\right) - \exp\left(-a_k D(\Delta + t_p)\right) \\ -4\exp\left(-a_k D(t_r + t_p)\right) + 2\exp\left(-a_k D(2t_r + t_p)\right) \\ +2\exp\left(-a_k D(\Delta - t_r - t_p)\right) + 2\exp\left(-a_k D(\Delta + t_r + t_p)\right) \\ -\exp\left(-a_k D(\Delta - 2t_r - t_p)\right) - \exp\left(-a_k D(\Delta + 2t_r + t_p)\right) \\ -4a_k D t_r + \frac{4}{3} a_k^3 D^3 t_r^3 + 2a_k^3 D^3 t_r^2 t_p + 4 \end{array} \right\} \tag{S3}$$

where t_r is the gradient rise time, t_p is the duration of each gradient plateau, and Δ is the separation of two diffusion gradients. Similarly, the analytical expression for the cosine-modulated trapezoidal OGSE sequences can also be derived from the Eq. (S2), these results have been shown previously (Xu J, et al. Magnetic resonance imaging of mean cell size in human breast tumors. Magn Reson Med 2020).

In this study, tumor cells were modeled as ideal spheres, then the coefficients B_k and a_k can be expressed as:

$$B_k = \frac{2(R/\mu_k)^2}{\mu_k - 2}, \qquad a_k = \left(\frac{\mu_k}{R}\right)^2 \tag{S4}$$

where μ_k is the kth root of $\mu J'_{3/2}(\mu) - \frac{1}{2} J_{3/2}(\mu) = 0$ and R is the tumor cell radius. For the intracellular diffusion, the restricted dMRI signal S_r can be expressed as:

$$S_r = S_0 \exp(-\phi) = S_0 \exp(-b \cdot \mathrm{ADC_r}) \tag{S5}$$

where b is the diffusion-weighted factor (b value) and $\mathrm{ADC_r}$ is the apparent restricted diffusion coefficient, then it can be calculated as:

$$\mathrm{ADC_r} = \frac{\phi}{b} \tag{S6}$$

Based on Eq. (S6) and (S3), we can obtain the analytical expression of $\mathrm{ADC_r}$, which is related to the cell diameter d or radius R, intracellular intrinsic diffusivity D_{in} ($D = D_{in}$), and specific diffusion-weighted sequences (PGSE and OGSE in this study).

IMPULSED neglects the transcytolemmal water exchange between the intra- and extracellular compartments, where tumor cells are modeled as impermeable ideal spheres. Then the dMRI signals are modeled as the sum of signals arising from the intra- and extracellular compartments:

$$S = S_{in} + S_{ex} \tag{S7}$$

where the water diffusion is restricted and hindered in the intra- and extracellular compartments, respectively, the corresponding signal attenuation is:

$$S_{in} = S_{in,0} \exp(-b \cdot \mathrm{ADC_r}) = v_{in} S_0 \exp(-b \cdot \mathrm{ADC_r}) \tag{S8}$$

and

$$S_{ex} = S_{ex,0} \exp(-b \cdot D_{\mathrm{ex}}) = v_{ex} S_0 \exp(-b \cdot D_{\mathrm{ex}}) \tag{S9}$$

where $S_{in,0}$ and $S_{ex,0}$ are non-diffusion-weighted signals, v_{in} and v_{ex} are the volume fractions of intra- and extracellular compartments, and D_{ex} is the extracellular hindered diffusivity. Note that $v_{in} + v_{ex} = 1$, the overall signal S can be expressed as:

$$S = S_0(v_{in} \cdot \exp(-b \cdot \mathrm{ADC_r}) + (1 - v_{in}) \cdot \exp(-b \cdot D_{\mathrm{ex}})) \tag{S10}$$

JOINT

The JOINT method is based on the Kärger model and the IMPULSED framework, which incorporated transcytolemmal water exchange. The magnetization exchange between the two compartments (S_{in} and S_{ex}) caused by water exchange can be quantified by the modified Kärger model:

$$\begin{aligned} \frac{dS_{in}}{dt} &= -\gamma^2 g^2 \delta^2 \cdot \mathrm{ADC_r} \cdot S_{in} - k_{in} S_{in} + k_{ex} S_{ex} \\ \frac{dS_{in}}{dt} &= -\gamma^2 g^2 \delta^2 \cdot D_{ex} \cdot S_{in} - k_{ex} S_{ex} + k_{in} S_{in} \end{aligned} \tag{S11}$$

where g and δ are the strength and duration of the diffusion gradient for the PGSE sequences, k_{in} and k_{ex} are the exchange rate constants of magnetizations (from 'in' to 'ex' and from 'ex' to 'in') and $k_{ex} = k_{in} v_{in} / v_{ex}$. By solving the differential Eq. (S11), the diffusion-weighted signal S can be expressed as the following linear combination of exponential terms:

$$S = S_{in} + S_{ex} = S_0(V_1 \exp(-bD_1^*) + (1 - V_1) \exp(-bD_2^*)) \tag{S12}$$

where the terms D_1^*, D_2^*, and V_1 are:

$$D_1^* = \frac{A_{in} - A_{ex} - D_Q}{2}$$
$$D_2^* = \frac{A_{in} - A_{ex} + D_Q}{2} \quad \text{(S13)}$$
$$V_1 = 1 - \frac{\left(A_{ex} - A_{in} + D_Q - 2k_{ex}/\gamma^2 g^2 \delta^2\right)v_{ex} + \left(A_{in} - A_{ex} + D_Q - 2k_{in}/\gamma^2 g^2 \delta^2\right)v_{in}}{2D_Q}$$

The other unknown parameters are computed by:

$$A_{in} = \mathrm{ADC_r} + \frac{k_{in}}{\gamma^2 g^2 \delta^2}$$
$$A_{ex} = D_{ex} + \frac{k_{ex}}{\gamma^2 g^2 \delta^2} \quad \text{(S14)}$$
$$D_Q = \sqrt{(A_{in} - A_{ex})^2 + \frac{4k_{in}k_{ex}}{\gamma^4 g^4 \delta^4}}$$

Substituting Eqs. (S14) and (S13) into (S12), we can obtain the final analytical expression after introducing the effect of transcytolemmal water exchange. Note that in the JOINT method, only the PGSE signals are expressed using Eq. (S12), while the other OGSE signals are still represented by Eq. (S10), since this time-dependent diffusion MRI method assumes that water exchange has a limited impact on signal acquisitions for the OGSE sequences with short diffusion times.

EXCHANGE

JOINT makes a strong approximation, i.e., transcytolemmal water exchange influences PGSE acquisitions only, not on OGSE. Due to this reason, numerical simulations and in vitro cell experiments have shown that the accuracy of the JOINT-derived parameters was only valid with relatively slow water exchange. The EXCHANGE method is also based on the Kärger model and IMPULSED analytical framework, but removes the approximation used in JOINT.

Specifically, first, a two-mode diffusion model is proposed to describe the actual intracellular diffusion, which includes not only restricted but also hindered diffusion in the presence of transcytolemmal water exchange. In this model, the probability of an intracellular molecule crossing the membrane and then moving to the extracellular space is defined as p, and it can be estimated from the existing parameters, including R, D_{in}, and k_{in}:

$$p = \frac{\left(\frac{4R}{3}\right)^2}{\left(\frac{4R}{3}\right)^2 + \frac{2D_{in}}{k_{in}} - \left(\frac{3R}{4}\right)^2} \quad \text{(S15)}$$

Then, the diffusion of intracellular water molecules is divided into two modes: for molecules that stay inside the cell, i.e., restricted diffusion, $\mathrm{ADC_r}$ is used to describe the intensity of the diffusion movement; For molecules that cross the membrane, leave the cell, and undergo hindered diffusion, an average hindered diffusivity D_{inh} is introduced and approximated as a linear combination of $\mathrm{ADC_r}$ and D_{ex}, with the volume fractions v_{in} and v_{ex} as the weights, i.e., $D_{inh} = v_{in}\mathrm{ADC_r} + v_{ex}D_{ex}$. The

two-mode diffusion coefficient of the intracellular compartment D_{in}^* can be calculated by the following approximation:

$$D_{in}^* = -\frac{\ln\big((1-p)\exp(-b\cdot \mathrm{ADC_r}) + p\exp(-b\cdot D_{inh})\big)}{b} \tag{S16}$$

Finally, replace the $\mathrm{ADC_r}$ in Eq. (S11) with D_{in}^*.

Second, a dimensional-analysis-based expression is used to correct the restriction-induced edge-enhancement effect. As shown in our previous work (Shi D, et al. Restriction-induced time-dependent transcytolemmal water exchange: Revisiting the Kärger exchange model. J Magn Reson 2024), the actual exchange rate constants of magnetizations k_{in}^m and k_{ex}^m are usually unequal to those of water molecules (k_{in} and k_{ex}), and typically $k_{in}^m > k_{in}$ (which is termed "edge-enhancement effect"). A modified form of k_{in}^m based on the dimensional analysis has been constructed:

$$k_{in}^m = k_{in}\cdot\left(1+\alpha\cdot(k_{in}bd^2)^{\gamma_1}\cdot\left(\frac{k_{in}d^2}{D_{in}}\right)^{\gamma_2}\cdot(v_{in})^{\gamma_3}\right) \tag{S17}$$

where the constants ($\alpha, \gamma_1, \gamma_2, \gamma_3$) are equal to (2.39, 0, 0.83, 2.88), (2.35,0.045,0.58, 3), and (1.7, 0.12, 0.48, 3) for the used PGSE, OGSE N=1 and N=2 sequences, respectively. On the other hand, the extracellular space is regarded as a narrow interstitial space, and there is an approximation that: $k_{ex}^m \approx k_{ex} = k_{in}v_{in}/v_{ex}$.

Third, a discretization-based computational framework is proposed to obtain the restricted dMRI signals and the corresponding $\mathrm{ADC_r}$ under arbitrary gradient waveforms, thus improving the adaptability of EXCHANGE. Based on the Eq. (S2) and discretization of the gradient waveform (**Figure S1**), the exponential attenuation factor ϕ can be expressed as:

$$\phi = \frac{\gamma^2}{2}\sum_k B_k \sum_{i=1}^{M}\sum_{j=1}^{M}\int_{t_{i-1}}^{t_i}\mathrm{d}t_1\int_{t_{j-1}}^{t_j}\mathrm{d}t_2\exp(-a_kD_{in}|t_2-t_1|)\,g(t_1)g(t_2) \tag{S18}$$

where $t_i = i\tau$, then a series of $M\times M$ symmetric matrices $\mathbf{C}^k$ are defined, whose elements are:

$$C_{ij}^k = \int_{t_{i-1}}^{t_i}\mathrm{d}t_1\int_{t_{j-1}}^{t_j}\mathrm{d}t_2\exp(-a_kD_{in}|t_2-t_1|)\,g(t_1)g(t_2) \tag{S19}$$

Each element C_{ij}^k can be calculated by the following approach: first, approximate $g(t)$ during each short pulse as:

$$g(t) \approx g\left(t_{i-1}+\frac{\tau}{2}\right), \text{for: } t_{i-1} < t < t_i \text{ (i.e. } t_{i-1}+\tau) \tag{S20}$$

Then for $i < j$:

$$\begin{aligned} C_{ij}^k = C_{ji}^k &= \int_{t_{i-1}}^{t_i} g(t_1)\exp(a_kD_{in}t_1)\,\mathrm{d}t_1\cdot\int_{t_{j-1}}^{t_j} g(t_2)\exp(-a_kD_{in}t_2)\,\mathrm{d}t_1 \\ &= \frac{2(\cosh(a_kD_{in}\tau)-1)}{(a_kD_{in})^2}\,g\left(t_{i-1}+\frac{\tau}{2}\right)g\left(t_{j-1}+\frac{\tau}{2}\right)\exp\left(-a_kD_{in}(t_j-t_i)\right) \end{aligned} \tag{S21}$$

And for $i = j$:

$$C_{ii}^{k} = \int_{t_{i-1}}^{t_i} dt_1 \int_{t_{i-1}}^{t_i} dt_2 \exp(-a_k D_{in}|t_2 - t_1|)\, g(t_1) g(t_2)$$

$$= \frac{2g\left(t_{i-1} + \frac{\tau}{2}\right)^2}{(a_k D_{in})^2}(a_k D_{in}\tau + \exp(-a_k D_{in}\tau) - 1) \quad \text{(S22)}$$

Based on Eqs. (S21) and (S22), the attenuation factor ϕ can be easily computed as:

$$\phi = \frac{\gamma^2}{2}\sum_k B_k \sum_{i=1}^{M}\sum_{j=1}^{M} C_{ij}^{k} = \frac{\gamma^2}{2}\sum_k B_k \mathrm{sum}(\mathbf{C}^k) \quad \text{(S23)}$$

where “sum” means to sum all matrix elements. In addition, the b-value can be expressed as:

$$b = \gamma^2 \int_0^{\mathrm{TE}} \left(\int_0^t g(t')\mathrm{d}t'\right)^2 \mathrm{d}t \quad \text{(S24)}$$

Then introduce an auxiliary function $f(t)$:

$$f(t) = \int_0^t g(t')\mathrm{d}t' \quad \text{(S25)}$$

Then the b-value can be rewritten as:

$$b = \gamma^2 \int_0^{\mathrm{TE}} f(t)^2 \mathrm{d}t = \gamma^2 \sum_{i=1}^{M} \int_{t_{i-1}}^{t_i} f(t)^2 \mathrm{d}t \approx \gamma^2 \sum_{i=1}^{M} \frac{\tau}{2}\left(f(t_{i-1})^2 + f(t_i)^2\right) \quad \text{(S26)}$$

where:

$$f(t_i) = f(i\tau) = \sum_{j=1}^{i} \int_{t_{j-1}}^{t_j} g(t')\mathrm{d}t' \approx \sum_{j=1}^{i} \tau g\left(t_{i-1} + \frac{\tau}{2}\right) \quad \text{(S27)}$$

Based on Eq. (S27), the b-value can be computed by Eq. (S26) for arbitrary gradient waveforms. Then the apparent restricted diffusion coefficient $\mathrm{ADC_r}$ can be calculated using Eq. (S6).

Simulation in silico, cells in vitro and animal studies have shown that EXCHANGE can provide accurate estimation of cell diameter d, intracellular volume fraction v_{in}, and water exchange rate constant k_{in} simultaneously.

Fig. S1 Discretization of arbitrary waveform.

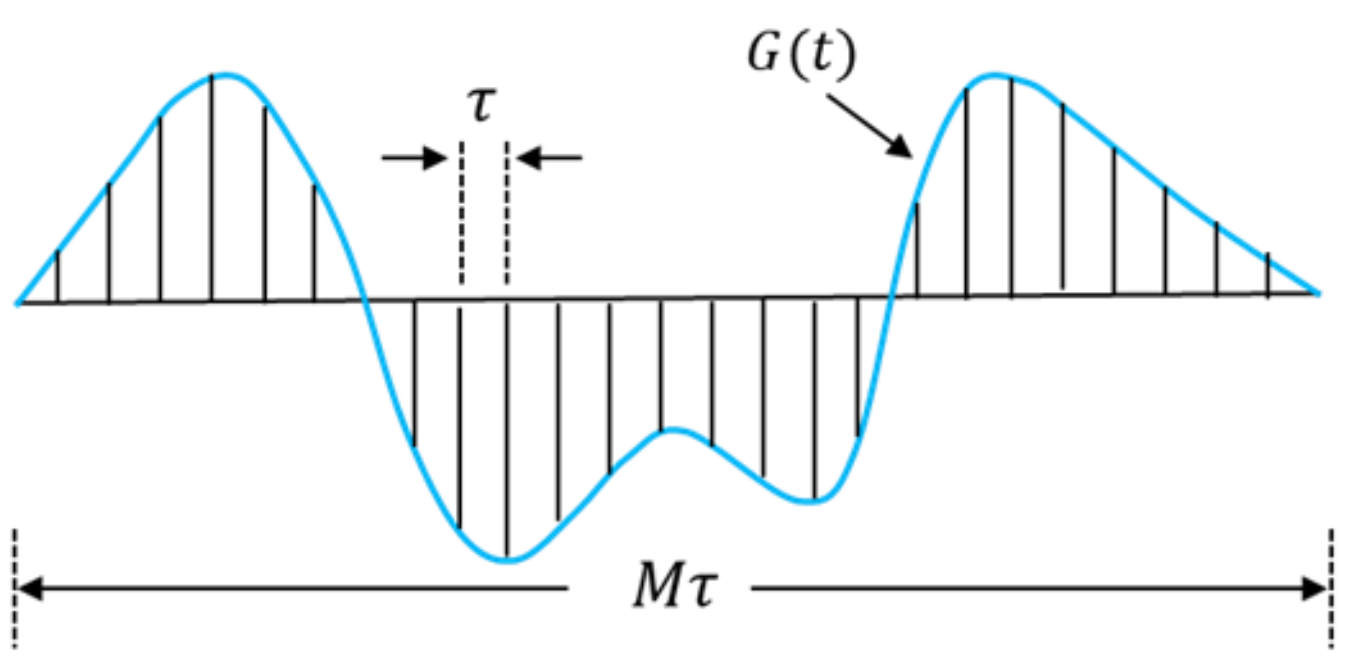

Appendix S2. Inter-and Intra-reader agreement assessments.

A subset of 40 patients was randomly selected from the full study population. To assess intra-reader reliability, tumor segmentations were repeated by Radiologist 1 at least 8 weeks after the initial segmentation. To assess inter-reader reliability, the second radiologist (Radiologist 2) independently performed the same segmentations using the previously described method. Intra-class correlation coefficients (ICCs) were then calculated to quantify both intra-reader and inter-reader agreement for all extracted quantitative parameters.
